# Cryolaser cryosclerotherapy versus isolated sclerotherapy for telangiectasias and reticular veins: A systematic review and meta-analysis

**DOI:** 10.1016/j.jvsv.2026.102582

**Published:** 2026-07-24

**Authors:** Gabriel Henrique Simoni, Dario Madera, Nikolaos Papatheodorou, Samantha Neves, Ana Clara Pizarro Varella, Julia Gonçalves Alonso, Nasser Mahfouz

**Affiliations:** a School of Medicine, University Center of Várzea Grande, Várzea Grande, Mato Grosso, Brazil; b Facultad de Medicina, Vascular Surgery Department, Universidad de la República (UDELAR), Montevideo, Uruguay; c Department of Vascular and Endovascular Surgery, Klinikum rechts der Isar, Technical University of Munich, Munich, Germany; d Faculdade de Ciências Médicas da Santa Casa de São Paulo, São Paulo, Brazil

**Keywords:** CLaCS, Cryolaser cryosclerotherapy, Meta-analysis, Reticular veins, Sclerotherapy, Telangiectasias

## Abstract

**Objective:**

To compare the incidence of adverse events between cryolaser cryosclerotherapy (CLaCS) and isolated sclerotherapy for lower limb telangiectasias and reticular veins and to determine pooled adverse event rates after CLaCS.

**Methods:**

PubMed, Embase, and Cochrane Central Register of Controlled Trials were searched from inception through February 2025 for studies of adults with telangiectasias or reticular veins (Clinical, Etiological, Anatomical, and Pathophysiological C1-C2) treated with CLaCS, reporting quantitative adverse event data. Risk ratios (RRs) were pooled using Mantel-Haenszel random-effects models for comparative studies. Single-arm proportions were pooled using Freeman-Tukey double arcsine transformation. Primary outcomes were telangiectatic matting, microthrombi formation, and hyperpigmentation. Secondary outcomes were ecchymosis and skin necrosis. An additional single-arm pooled proportion of satisfactory vessel clearance was estimated as a secondary efficacy outcome.

**Results:**

Six studies (833 patients) were included; three provided direct comparative data (363 CLaCS vs 259 sclerotherapy). CLaCS was associated with significantly lower rates of matting (RR, 0.07 [95% confidence interval [CI], 0.01-0.36]), microthrombi (RR, 0.18 [95% CI, 0.06-0.54]), and ecchymosis (RR, 0.43 [95% CI, 0.32-0.58]). No significant difference was found for hyperpigmentation (RR, 0.29 [95% CI, 0.06-1.44]; I^2^ = 92.1%) or skin necrosis (RR, 0.13 [95% CI, 0.02-1.00]). Pooled single-arm rates after CLaCS were 0.00% for matting, 0.00% for skin necrosis, 6.31% for microthrombi, 7.38% for hyperpigmentation, and 22.41% for ecchymosis. The pooled single-arm rate of satisfactory vessel clearance after CLaCS was 90.68% (95% CI, 76.74-98.87; I^2^ = 94.6%) across five studies. Patient satisfaction, reported in three studies and synthesized narratively because of instrument heterogeneity, was consistently high (∼85%-90%) across techniques.

**Conclusions:**

CLaCS was associated with significantly lower rates of matting, microthrombi, and ecchymosis compared with isolated sclerotherapy, with near-zero incidence of matting and skin necrosis. High heterogeneity in hyperpigmentation highlights the need for trials with standardized outcome measures. CLaCS also achieved a high pooled rate of satisfactory vessel clearance and consistently high patient satisfaction across studies, supporting its efficacy alongside its favorable safety profile.

**Article Highlights**

• **Type of Research:** Systematic review and meta-analysis of randomized and nonrandomized studies comparing cryolaser cryosclerotherapy (CLaCS) with isolated sclerotherapy for lower limb telangiectasias and reticular veins
• **Key Findings:** In six studies encompassing 833 patients, CLaCS was associated with significantly lower rates of telangiectatic matting (risk ratio [RR], 0.07; 95% confidence interval [CI], 0.01-0.36), microthrombi formation (RR, 0.18; 95% CI, 0.06-0.54), and ecchymosis (RR, 0.43; 95% CI, 0.32-0.58) compared with isolated sclerotherapy. Pooled single-arm incidence after CLaCS was 0.00% for matting, 0.00% for skin necrosis, 6.31% for microthrombi, 7.38% for hyperpigmentation, and 22.41% for ecchymosis. No statistically significant difference between CLaCS and isolated sclerotherapy was found for hyperpigmentation (RR, 0.29; 95% CI, 0.06-1.44) or skin necrosis (RR, 0.13; 95% CI, 0.02-1.00).
• **Take Home Message:** CLaCS offers a favorable adverse event profile compared with isolated sclerotherapy, with near-zero rates of matting and skin necrosis, supporting its role as a safer alternative in patients with higher Fitzpatrick skin phototypes and elevated aesthetic expectations.

Chronic venous disease (CVD) is among the most prevalent vascular conditions worldwide, affecting 15% to 80% of the adult population depending on the diagnostic criteria and population studied.^1^^,^^2^ Telangiectasias and reticular veins, classified as C1 under the Clinical, Etiological, Anatomical, and Pathophysiological (CEAP) system,^3^ represent the earliest and most common clinical manifestation^4^ and account for the majority of patients seeking treatment in aesthetic phlebology practice.^5^

Sclerotherapy remains the recommended first-line treatment for telangiectasias and reticular veins according to international guidelines, including those from the European Society for Vascular Surgery (ESVS)^6^ and the Society for Vascular Surgery/American Venous Forum.^7^ Both liquid and foam formulations of detergent sclerosants (polidocanol and sodium tetradecyl sulfate) and hyperosmolar agents (hypertonic dextrose) are widely employed.^8^, ^9^, ^10^ Despite established efficacy, sclerotherapy is associated with well-documented adverse events that may compromise cosmetic outcomes and patient satisfaction.^11^ Postsclerotherapy hyperpigmentation (PSH) occurs in 10% to 40% of treated patients and may persist for months.^12^^,^^13^ Telangiectatic matting, the development of new fine telangiectasias at or near the treatment site, has been reported in up to 33% of cases.^14^^,^^15^ Microthrombi formation with a subsequent need for drainage is another common sequela that contributes to inflammation, hemosiderin deposition, and ultimately PSH.^12^^,^^13^

The integration of transdermal laser therapy with sclerotherapy has emerged as a strategy to enhance treatment efficacy while mitigating adverse events.^16^ Cryolaser cryosclerotherapy (CLaCS), first described by Miyake in the late 1990s,^17^^,^^18^ combines three elements: (1) transdermal Nd:YAG 1064-nm laser emission directed at the target vessel, (2) cryoanalgesia via skin cooling devices to prevent epidermal damage and reduce pain, and (3) injection of a mild sclerosing agent (typically 75% hypertonic dextrose) into the vessel previously damaged by laser absorption.^19^ The rationale is synergistic: the laser causes endothelial damage, tunica media edema, and vessel diameter reduction before the sclerosant is injected, thereby requiring lower sclerosant volumes and concentrations while achieving effective vessel obliteration.^19^^,^^20^ The high viscosity of hypertonic dextrose prevents passage into capillary and arteriolar networks under typical injection pressures, reducing the risk of cutaneous necrosis.^21^ This approach has been incorporated into the 2022 ESVS Clinical Practice Guidelines on the management of CVD.^6^

Despite growing adoption in over 30 countries,^22^ the comparative evidence supporting CLaCS over conventional sclerotherapy has not been systematically synthesized. Individual studies have reported favorable safety profiles,^19^^,^^23^, ^24^, ^25^, ^26^ but sample sizes have been modest and outcome definitions heterogeneous. A recent Cochrane review on treatments for telangiectasias and reticular veins identified laser plus sclerotherapy as a potentially superior approach but noted the paucity of comparative data.^11^ To our knowledge, no systematic review with meta-analysis has been conducted to quantitatively assess the safety of CLaCS compared with isolated sclerotherapy.

The objective of this systematic review and meta-analysis was twofold: (1) to compare the incidence of adverse events between CLaCS and isolated sclerotherapy using pooled risk ratios (RRs) from comparative studies and (2) to determine the overall pooled rates of adverse events associated with CLaCS from all available studies.

## Methods

This systematic review and meta-analysis was conducted in accordance with the Preferred Reporting Items for Systematic Reviews and Meta-Analyses 2020 guidelines.^27^ The protocol was registered in PROSPERO (CRD420261354282).

### Search strategy

A comprehensive literature search was performed in PubMed/MEDLINE, Embase, and Cochrane Central Register of Controlled Trials from database inception through February 2026. The search strategy combined terms related to the intervention (cryo-laser cryo-sclerotherapy, CLaCS, CLACS, LÁSTIK, cryo-sclerotherapy, and laser sclerotherapy), the target condition (telangiectasia, reticular veins, spider veins, and CEAP C1), and the comparator (sclerotherapy, polidocanol, sodium tetradecyl sulfate, and dextrose). Reference lists of included studies and relevant review articles were manually screened for additional eligible publications. No language or publication date restrictions were applied. The complete, line-by-line search strategy for each database, including all search terms and Boolean operators, is provided in the Appendix (online only) to permit replication and future updating of this meta-analysis.

### Eligibility criteria

#### Population

Adults (≥18 years) with telangiectasias and/or reticular veins of the lower limbs, classified as CEAP C1.^3^ Studies including mixed populations of C1 and C2 low (varicose veins of 3-5 mm) were accepted when the combined laser-sclerotherapy technique was the primary focus and adverse event data were reported for the pooled cohort (Table I).

**Table I:** Characteristics of included studies

Study	Country	Design	N	Population	Intervention	Comparator	Laser parameters	Follow-up
Rodrigues et al, 2025^24^	Brazil	RCT, within-patient, and blinded evaluators	23 pts (46 limbs)	Women ≥18 years; reticular veins ≤2.2 mm; C1; FP III-V	Nd:YAG 1064 nm + D75 + skin cooling + AR	0.5% POL foam; contralateral limb	6 mm spot; 15 ms; 70 J/cm^2^ (FP ≤ IV); 50 J/cm^2^ (FP V)	D7, D30, and D60
Nasser et al, 2024^23^	Egypt	RCT, parallel groups, and blinded evaluator	195/197	Women 18-70 years; C1; vein <1.5 mm; FP II-IV	Nd:YAG 1064 nm + D70 + Zimmer Cooler + AR	0.5% POL foam 8-10 mL/session	6 mm spot; 15 ms; 50-70 J/cm^2^	8 weeks postsession; 6 months total
Fonseca et al, 2023^25^	Brazil	RCT, triple-blind, single-center	49 patients (55 limbs); 51 limbs analyzed (26/25) (both CLaCS)	Women 18-70 years; C1; FP I-III	CLaCS G1: Nd:YAG + D75	CLaCS G2: Nd:YAG + POL 0.3% + D67 (internal comparator)	6 mm spot; 15 ms; 70 J/cm^2^; forced air cooling	30 days
Rosukhovskiy, 2021^26^	Russia	Prospective comparative, nonrandomized, 2-center	147/41	C1-C2; veins ≤4 mm; 96% women; mean age, ∼43 years	Nd:YAG 1064 nm microsecond + STS 0.3% in 40% glucose + contact cooling	Compression microsclerotherapy: same sclerosant, no laser	650-1500 μs; 50-90 J/cm^2^; spot 2-5 mm; contact cooling	1-2 weeks; 6-10 weeks
Miyake, 2014^28^	Brazil	Retrospective case series	140 (single arm)	CEAP C1-C2; 98% women; mean age 37 years	Nd:YAG 1064 nm + D75 + Cryo5 (−20°C) + AR	None	6 mm spot; 15 ms; up to 80 J/cm^2^	Multiple sessions (2-4-week intervals)
Sathler de Melo and Faccini, 2026^29^	Brazil	Retrospective cohort, single-center	41 pts; 71 sessions (single arm)	Women; C1+C2 low; age 23-79 years (median, 52.2); FP I-V	HHLCS: Nd:YAG 1064 nm + cooled D67-75% + Siberian cooling + VeinViewer	None	Spot 3 mm (150-200 J/cm^2^), 6 mm (60-90 J/cm^2^), and 9 mm (50-60 J/cm^2^)	3-12 months

*AR*, Augmented reality; *C1*, CEAP clinical class 1 (telangiectasias/reticular veins); *C2*, CEAP clinical class 2 (varicose veins); *CLaCS*, cryolaser cryosclerotherapy; *D67/D70/D75*, hypertonic dextrose 67%/70%/75%; *FP*, Fitzpatrick phototype; *HHLCS*, hemodynamic hybrid laser cryosclerotherapy; *Nd:YAG*, neodymium-doped yttrium aluminum garnet; *NR*, not reported; *POL*, polidocanol; *pts*, patients; *RCT*, randomized controlled trial; *STS*, sodium tetradecyl sulfate.

#### Intervention

CLaCS or equivalent combined techniques defined by three components: (1) transdermal Nd:YAG 1064-nm laser, (2) skin cooling (cryoanalgesia), and (3) adjunctive low-aggressiveness sclerotherapy.^19^ Variations in nomenclature (CLaCS, CLACS, LÁSTIK, and HHLCS) were accepted, provided the described technique contained all three elements.

#### Comparator

For comparative analyses, isolated sclerotherapy without laser, including liquid microsclerotherapy and foam sclerotherapy with any sclerosant,^8^ with or without compression therapy. Single-arm CLaCS studies without a comparator were included in the proportional meta-analysis.

#### Outcomes

Outcomes were defined a priori. Studies were required to report at least one predefined adverse event with quantifiable data. Primary outcomes were postprocedure hyperpigmentation, telangiectatic matting, and microthrombi (intravascular coagula). Secondary outcomes were ecchymosis (bruising) and skin necrosis. The three primary outcomes were prespecified as primary because they are the adverse events most consistently reported across the included studies, are the principal determinants of the final aesthetic result and of patient dissatisfaction after treatment of telangiectasias, and are mechanistically central to the hypothesized advantage of CLaCS, being directly linked to the sclerosant-induced inflammatory and thrombotic cascade. Ecchymosis and skin necrosis were prespecified as secondary because ecchymosis, although frequent, is typically transient and self-limiting and does not affect the final aesthetic outcome, whereas skin necrosis is rare and reported inconsistently across studies. Microthrombi (also termed intravascular coagula or trapped intraluminal blood) were defined as the presence of coagulated blood within a treated vessel detected on clinical examination during follow-up, irrespective of whether drainage (needle thrombectomy or aspiration) was required; when a primary study reported only coagula that required drainage, that count was extracted, and this distinction is noted per study in Table II. All outcome definitions applied uniformly across the comparative and single-arm analyses are operationally specified in Table III.

**Table II:** Adverse event outcomes by study

Study	Hyperpigmentation	Telangiectatic matting	Microthrombi/Need for drainage	Skin necrosis	Ecchymosis	Vessel clearance	Patient satisfaction
Rodrigues et al, 2025^24^	Photo: CLaCS 7/21 (33.3%) vs foam 5/21 (23.8%); *P* = .89. Colorimetry ΔE intensity: CLaCS 1.30 vs foam 1.44; *P* = .027	CLaCS 0/21 (0%) vs foam 1/21 (4.7%); *P* = 1.0	CLaCS 3/23 (13.0%) vs foam 9/23 (39.1%); *P* = .035	0 in both arms	CLaCS 3/23 (13.0%) vs foam 9/23 (39.1%); *P* = .016	NR	ANA scale (0-10): CLaCS pre 4.48 → post 9.48 (*P* < .001); foam pre 4.62 → post 9.14 (*P* < .001); no difference between techniques
Nasser et al, 2024^23^	CLaCS 16/195 (8.2%) vs sclero 172/197 (87.3%); *P* < .001. At 6 months, sclero rate decreased to 45%	CLaCS 0/195 (0%) vs sclero 38/197 (19.3%); *P* < .001	CLaCS 14/195 (7.2%) vs sclero 72/197 (36.5%); *P* < .001	CLaCS 1/195 (0.5%) vs sclero 8/197 (4.1%); *P* = .019	CLaCS 39/195 (20.0%) vs sclero 90/197 (45.7%); *P* < .001	Complete elimination: 1st session G-A 64.6% vs G-B 50.3% (*P* = .004); 2nd session 86.2% vs 74.1%; and 3rd session 100% vs 85.3% (*P* < .001)	NR
Fonseca et al, 2023^25^	D75: 2/26 (7.7%) vs POL + D67: 4/25 (16.0%); *P* = .399. Pooled CLaCS: 6/51 (11.8%)	0 in both groups	NR	0 in both groups	NR	Photographs without AR (30 days): G1 excellent 65.4%/good 19.2%/fair 3.8%/poor 11.5% vs G2 76%/4%/0%/20% (*P* = .613).	Excellent 27.5%/very good 58.8%/good 11.8%/fair 2%/ineffective 0% (no difference between groups)
Rosukhovskiy, 2021^26^	CLaCS ∼5% (7/147) vs CM ∼22% (9/41); *P* = .006	Severe matting: CLaCS 2/147 (1.4%) vs CM 9/41 (22.0%); *P* < .0001	CLaCS 0/147 (0%) vs CM 11/41 (26.8%)	0 in both groups	NR	Five-point efficacy scale: 1-2 weeks CLaCS 3.9 ± 0.6 vs CM 2.3 ± 0.8 (*P* < .0001); 6-10 weeks CLaCS 4.5 ± 0.4 vs CM 3.8 ± 0.7 (*P* < .0001). Reduction >75%: CLaCS 22.4% vs CM 0.5%	NR
Miyake, 2014^28^	0/140 (0%)	0/140 (0%)	Intravascular coagula: ∼20/140 (14.3%)	0/140 (0%)	∼42/140 (30.0%)	Partial/complete improvement with patient satisfaction: 86% (121/140); no response or worsening: 14% (19/140)	Patient's subjective assessment integrated with clearance outcome (86% satisfied)
Sathler de Melo and Faccini, 2026^29^	Most common complication; all resolved spontaneously; exact count NR	NR	NR	0/41 (0%)	NR	Complete clearance (≥70%): 83% (34/41)	NR

*CLaCS*, Cryolaser cryosclerotherapy; *CM*, compression microsclerotherapy; *D67/D75*, hypertonic dextrose 67%/75%; *NR*, not reported; *POL*, polidocanol; *sclero*, sclerotherapy; *ΔE*, CIELAB color difference.

**Table III:** Operational definitions of study outcomes

Outcome	Classification	Operational definition	Ascertainment notes
Postprocedure hyperpigmentation	Primary	Persistent brown cutaneous discoloration along or around treated vessels after treatment, attributed to hemosiderin and/or melanin deposition	Assessed by photographic, clinical, blinded-evaluator, or objective colorimetric (CIELAB ΔE) methods, varying by study; when only qualitatively described without a count, it is not included in the pooled estimate
Telangiectatic matting	Primary	Appearance of new, fine (<0.2 mm) telangiectasias at or near the treated site after treatment	Clinical and/or photographic assessment
Microthrombi (intravascular coagula)	Primary	Coagulated blood trapped within a treated vessel, detected on clinical examination during follow-up, whether or not drainage (needle thrombectomy or aspiration) was required	Where a primary study reported only coagula that required drainage, that count was extracted; the reporting threshold is noted per study in Table II
Ecchymosis (bruising)	Secondary	Visible extravasation of blood into the skin or subcutis (bruising) at treated sites, typically self-limiting	Clinical and/or photographic assessment
Skin necrosis	Secondary	Partial- or full-thickness cutaneous tissue death (ulceration) at a treated site	Clinical assessment
Satisfactory vessel clearance	Secondary (efficacy)	An “excellent” or “complete” clearance response as defined by each primary study, evaluated at the last reported follow-up	Operationalized differently across studies (blinded photographic grading, 5-point efficacy scale, binary lesion elimination, or a categorical ≥70% reduction threshold)

*CIELAB*, International Commission on Illumination L∗a∗b∗ color space; *CLaCS*, cryolaser cryosclerotherapy; *ΔE*, CIELAB color difference.

#### Study design

Randomized controlled trials (RCTs), nonrandomized comparative studies, prospective or retrospective cohort studies, and case series with a minimum of 10 patients. Case reports, narrative reviews, editorials, letters, and conference abstracts were excluded. When duplicate publications from the same cohort were identified, the most recent or comprehensive report was retained.

### Study selection and data extraction

Two reviewers independently screened titles and abstracts, followed by full-text review of potentially eligible studies. Disagreements were resolved by consensus. Data were extracted using a standardized form that captured study characteristics (authors, year, country, design, sample size, population, CEAP classification, and Fitzpatrick skin types^30^), intervention details (laser parameters, sclerosant type and concentration, and cooling method), comparator details, follow-up duration, and outcome data (number of events and denominators for each predefined adverse event). When outcomes were reported only as percentages, absolute event counts were calculated from the reported percentage and total sample size.

### Risk-of-bias assessment

Risk of bias (RoB) was assessed using the Cochrane RoB 2 tool^31^ for RCTs and the Risk of Bias in Nonrandomized Studies of Interventions (ROBINS-I) tool^32^ for the nonrandomized comparative study (Rosukhovskiy^26^). For single-arm studies without a comparator group (Miyake^28^; Sathler de Melo and Faccini^29^), methodological quality was appraised using the Joanna Briggs Institute Critical Appraisal Checklist for Case Series. Assessments were performed independently by two reviewers, with discrepancies resolved by discussion. Results were summarized in a plot and used descriptively to contextualize the certainty of findings; no studies were excluded based on RoB.

### Statistical analysis

Two complementary meta-analytic strategies were employed. First, for the primary analysis, comparative meta-analyses were performed using data from studies that directly compared CLaCS with isolated sclerotherapy. RRs with 95% confidence intervals (CIs) were calculated for each adverse event outcome. Pooled estimates were obtained using the Mantel-Haenszel method^33^ under a random-effects model with the DerSimonian-Laird estimator for between-study variance (τ^2^).^34^ Several outcomes were sparse, with zero events in one or both arms of individual studies. To avoid discarding these informative studies, they were retained, and a continuity correction of 0.5 was applied to all cells of any study containing a zero cell, in keeping with the default handling of such data by the Mantel-Haenszel method.^35^ Because the Mantel-Haenszel approach with a fixed continuity correction can bias effect estimates when events are rare, pooled RRs for the sparse outcomes (matting, microthrombi, and skin necrosis) were regarded as approximate and were interpreted alongside the corresponding single-arm proportion estimates, with the concordance between the two approaches used to gauge robustness.^35^^,^^36^ Both common-effect and random-effects estimates were computed; the random-effects model was considered the primary analysis, given the anticipated clinical and methodological heterogeneity across studies. Patient satisfaction and the comparative efficacy of CLaCS vs isolated sclerotherapy on vessel clearance were synthesized narratively rather than meta-analytically, owing to substantial methodological heterogeneity in assessment instruments, scales, time points, and study designs across the studies reporting these outcomes.

Second, single-arm meta-analyses of proportions were conducted to estimate the pooled incidence of each adverse event in CLaCS-treated patients across all included studies. Proportions were pooled using the Freeman-Tukey double arcsine transformation^37^ to stabilize variance, particularly for proportions near zero. Individual study CIs were calculated using the Clopper-Pearson exact method.^38^ A random-effects model with DerSimonian-Laird estimation was used for all pooled proportion estimates. Satisfactory vessel clearance was also analyzed under this same framework as a secondary efficacy outcome; for the purposes of pooling, a satisfactory clearance event was defined a priori as the equivalent of an “excellent” or “complete” clearance response as reported in each primary study, at the last reported follow-up. The single-arm meta-analysis of proportions is a descriptive synthesis of within-group event rates and is methodologically distinct from the comparative analysis: single-arm CLaCS studies contribute only to these pooled incidence estimates and never enter the comparative risk-ratio models, so their inclusion cannot bias the between-group comparisons. Because single-arm estimates lack an internal comparator and are therefore susceptible to between-study heterogeneity and to differences in populations, protocols, and outcome ascertainment, they were pooled under a random-effects model, reported with I^2^ and τ^2^, and interpreted as hypothesis-generating descriptive rates rather than as estimates of comparative effect.

Statistical heterogeneity was assessed using the Cochran *Q* test and quantified with the I^2^ statistic.^39^ I^2^ values were interpreted as low (<25%), moderate (25%-75%), or high (>75%).^39^ The τ^2^ statistic was reported as an absolute measure of between-study variance. Publication bias assessment through funnel plots was not performed owing to the limited number of studies per outcome (<10).^40^

All statistical analyses were conducted using R software (version 4.3.2; R Foundation for Statistical Computing) with the “meta” package (version 8.0.2).^41^ Statistical significance was defined as a two-sided *P* value less than .05. For the multiarm trial by Fonseca et al,^25^ the two CLaCS arms (CLaCS with 75% dextrose and CLaCS with 0.3% polidocanol plus 67% dextrose) were combined into a single CLaCS unit of analysis, in line with the recommendations of the Cochrane Handbook (Section 23.3.4) for multiarm trials in which the experimental arms represent variations of the same intervention.

## Results

### Study selection

**Fig 1:**
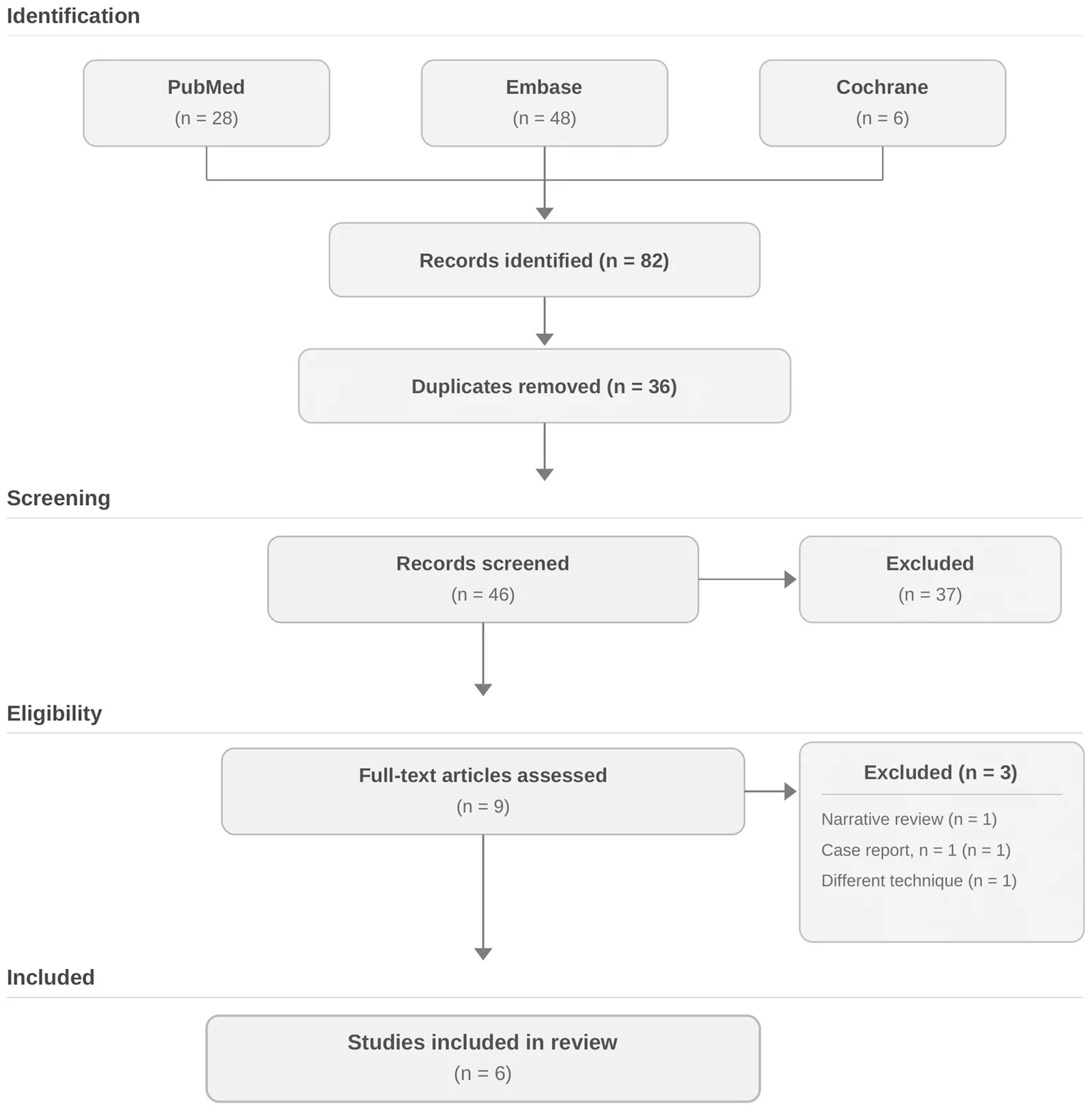
Preferred Reporting Items for Systematic Reviews and Meta-Analyses 2020 flow diagram of the systematic literature search and study selection.

The systematic search identified 82 records across three databases: PubMed (n = 28), Embase (n = 48), and Cochrane Central Register of Controlled Trials (n = 6). After removal of 36 duplicates, 46 unique records were screened by title and abstract. Of these, 37 were excluded for not meeting the eligibility criteria, and nine full-text articles were assessed for eligibility. Three studies were excluded after full-text review: one was a technical description and narrative review without an original cohort, one was a case report of a single patient, and one was a case report using a fundamentally different technique. Six studies met all inclusion criteria and were included in the qualitative synthesis and meta-analysis (Fig 1).^23^, ^24^, ^25^, ^26^

### Study characteristics

The six included studies^23^, ^24^, ^25^, ^26^^,^^28^^,^^29^ were published between 2014 and 2026 and encompassed a total of 833 patients or treated limbs (Table I). Three were RCTs,^23^, ^24^, ^25^ one was a prospective nonrandomized comparative study,^26^ and two were single-arm retrospective cohorts.^28^^,^^29^ Three studies provided direct comparative data between CLaCS and isolated sclerotherapy, totaling 363 CLaCS-treated and 259 sclerotherapy-treated patients or limbs.^23^^,^^24^^,^^26^

Studies were conducted in Brazil (n = 4), Egypt (n = 1), and Russia (n = 1). The populations were predominantly female (96%-100%) with CEAP classifications ranging from C1 to C2 low and Fitzpatrick skin phototypes I through V. All CLaCS interventions employed the Nd:YAG 1064-nm laser with fluences of 50 to 200 J/cm^2^, spot sizes of 2 to 9 mm, and pulse durations of 0.65 to 50 milliseconds.^16^ Sclerosants in the CLaCS arms included hypertonic dextrose (5 studies) and sodium tetradecyl sulfate 0.3% diluted in 40% glucose (1 study). In the comparator arms, 0.5% polidocanol foam was used in two studies^23^^,^^24^ and the same sclerosant without laser in one study.^26^ Follow-up periods ranged from 30 days to 12 months. Detailed study characteristics are presented in Table I.

### Comparative meta-analysis: CLaCS vs isolated sclerotherapy

Three studies (Rodrigues et al^24^; Nasser et al^23^; and Rosukhovskiy^26^) provided direct comparative data between CLaCS and isolated sclerotherapy, encompassing 363 CLaCS-treated and 259 sclerotherapy-treated patients or limbs.

#### Telangiectatic matting

**Fig 2:**
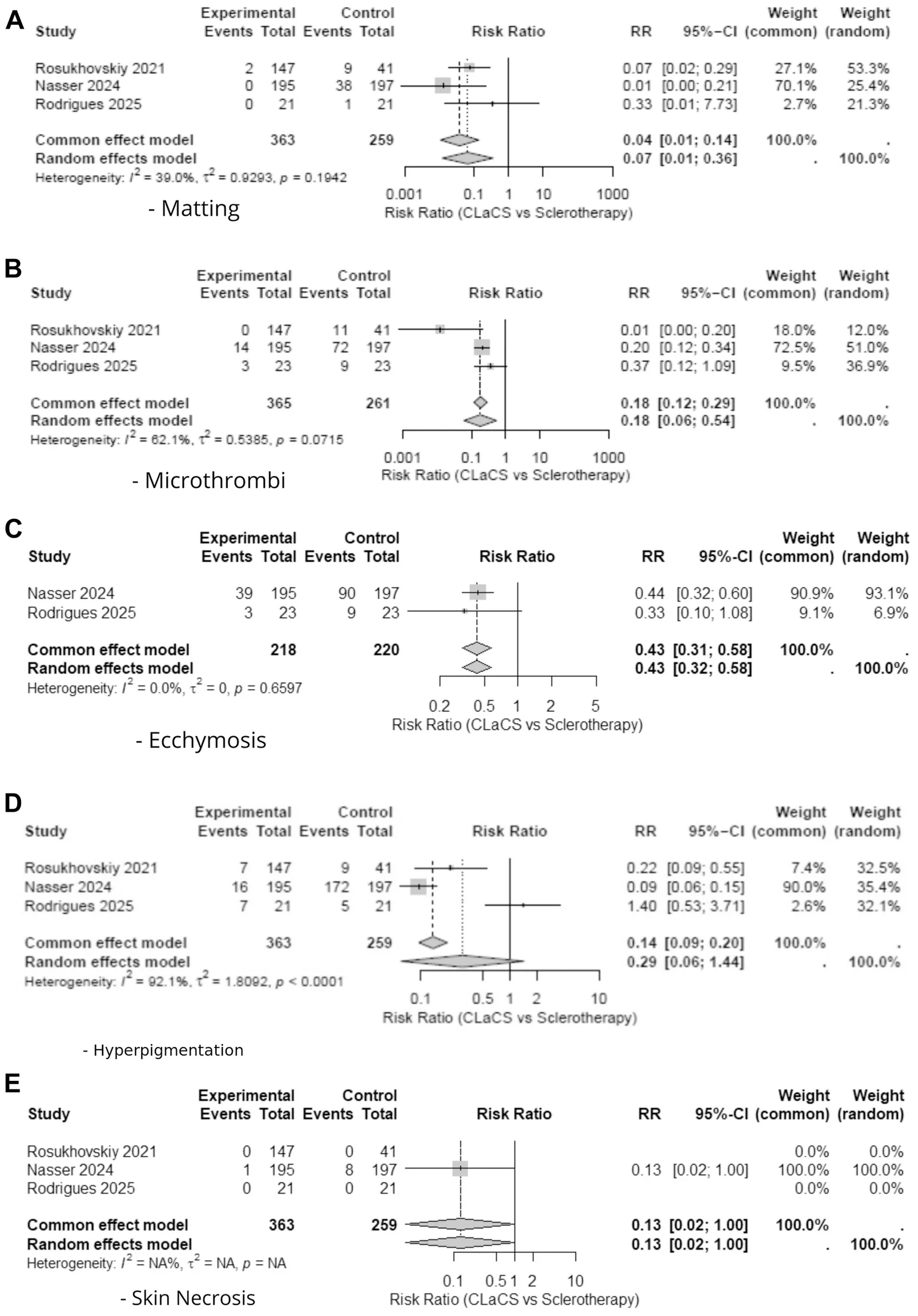
Forest plots of comparative meta-analyses (CLaCS vs isolated sclerotherapy) using Mantel-Haenszel random-effects models. **A,** Telangiectatic matting. **B,** Microthrombi formation or need for drainage. **C,** Ecchymosis. **D,** Hyperpigmentation. **E,** Skin necrosis. *CI*, Confidence interval; *CLaCS*, cryolaser cryosclerotherapy; *I*^*2*^, inconsistency statistic; *RR*, risk ratio; *τ*^*2*^, between-study variance.

CLaCS was associated with a significantly lower risk of telangiectatic matting compared with isolated sclerotherapy (RR, 0.07; 95% CI, 0.01-0.36; *P* < .01). Heterogeneity was low to moderate (I^2^ = 39.0%; τ^2^ = 0.9293; *P* = .19). Matting rates in the CLaCS arms ranged from 0% to 1.4%, compared with 4.8% to 22.0% in the sclerotherapy arms. In two of three studies, zero matting events were observed in the CLaCS group (Fig 2, *A*).

#### Microthrombi (intravascular coagula)

CLaCS was associated with a significantly lower risk of microthrombi (RR, 0.18; 95% CI, 0.06-0.54; *P* < .01). Heterogeneity was moderate (I^2^ = 62.1%; τ^2^ = 0.5385; *P* = .07). Microthrombi rates in the CLaCS arms ranged from 0% to 13.0%, compared with 26.8% to 39.1% in the sclerotherapy arms. Rosukhovskiy^26^ reported zero microthrombi requiring aspiration in the CLaCS group vs 26.8% in the sclerotherapy group (Fig 2, *B*).

#### Ecchymosis

Two studies (Nasser et al^23^ and Rodrigues et al^24^) reported ecchymosis as a separate outcome. CLaCS was associated with significantly less ecchymosis than sclerotherapy (RR, 0.43; 95% CI, 0.32-0.58; *P* < .001), with no detectable heterogeneity (I^2^ = 0.0%; τ^2^ = 0; *P* = .66). Ecchymosis rates were 13.0% to 20.0% in CLaCS arms vs 39.1% to 45.7% in sclerotherapy arms (Fig 2, *C*).

#### Hyperpigmentation

Under the random-effects model, no statistically significant difference in hyperpigmentation was found between CLaCS and sclerotherapy (RR, 0.29; 95% CI, 0.06-1.44; *P* = .13). However, substantial heterogeneity was present (I^2^ = 92.1%; τ^2^ = 1.8092; *P* < .0001). The direction of effect was inconsistent: Rosukhovskiy^26^ and Nasser et al^23^ reported markedly lower hyperpigmentation with CLaCS (RR, 0.22 and 0.09, respectively), whereas Rodrigues et al^24^ found a nonsignificant trend toward higher hyperpigmentation with CLaCS on photographic evaluation (RR, 1.40; 95% CI, 0.53-3.71). Notably, in the same study, colorimetric analysis demonstrated significantly lower intensity of pigmentation in the CLaCS group (ΔE, 1.30 vs 1.44; *P* = .027; Fig 2, *D*). The common-effect estimate was significant (RR, 0.14; 95% CI, 0.09-0.20).

#### Skin necrosis

Skin necrosis was a rare event. Only Nasser et al^23^ contributed informative data, with one event (0.5%) in the CLaCS group vs eight events (4.1%) in the sclerotherapy group. The pooled estimate showed a trend favoring CLaCS (RR, 0.13; 95% CI, 0.02-1.00), with the CI reaching the boundary of statistical significance. Rosukhovskiy^26^ and Rodrigues et al^24^ reported zero events in both arms (Fig 2, *E*).

### Single-arm meta-analysis: Pooled adverse event rates after CLaCS

Pooled adverse event rates were estimated from all CLaCS-treated patients across the six included studies using single-arm meta-analysis of proportions. The number of studies contributing data varied by outcome, depending on the availability of quantifiable event data.

#### Telangiectatic matting

**Fig 3:**
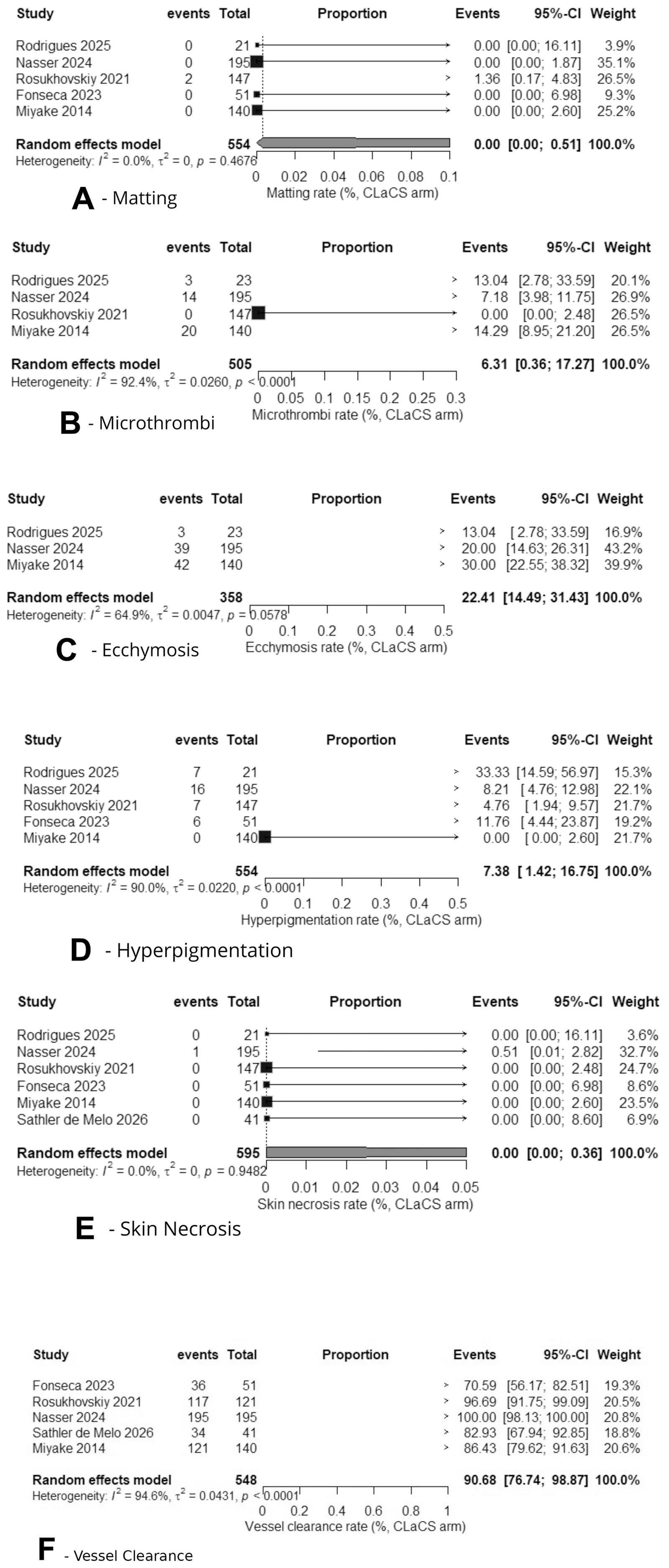
Forest plots of single-arm meta-analyses of proportions in CLaCS-treated patients using Freeman-Tukey double arcsine transformation and DerSimonian-Laird random-effects estimation. **A,** Telangiectatic matting. **B,** Skin necrosis. **C,** Microthrombi and intravenous coagula. **D,** Hyperpigmentation. **E,** Ecchymosis. **F,** Satisfactory vessel clearance. *CI*, Confidence interval; *CLaCS*, cryolaser cryosclerotherapy; *I*^*2*^, inconsistency statistic.

Five studies reported matting data. The pooled rate was 0.00% (95% CI, 0.00-0.51), with no heterogeneity (I^2^ = 0.0%; τ^2^ = 0). Four of five studies reported zero matting events; the only study with events (Rosukhovskiy^26^) reported 2 cases of severe matting among 147 patients (1.4%) (Fig 3, *A*).

#### Skin necrosis

All six studies contributed data. The pooled rate was 0.00% (95% CI, 0.00-0.36), with no heterogeneity (I^2^ = 0.0%; τ^2^ = 0). Five of six studies reported zero necrosis events. The single event occurred in the study by Nasser et al^23^: a 1- to 2-mm area managed conservatively without significant scarring (Fig 3, *B*).

#### Microthrombi (intravascular coagula)

Four studies reported data on microthrombi (intravascular coagula). The pooled rate was 6.31% (95% CI, 0.36-17.27), with high heterogeneity (I^2^ = 92.4%; τ^2^ = 0.0260). Individual study rates ranged from 0% (Rosukhovskiy^26^) to 14.3% (Miyake^28^), reflecting differences in the threshold for reporting and the distinction between coagula identified on examination and those requiring active drainage (Fig 3, *C*).

#### Hyperpigmentation

Five studies reported quantifiable hyperpigmentation data. Sathler de Melo and Faccini^29^ described hyperpigmentation as the most common complication but did not provide an exact count and was therefore excluded from this analysis. The pooled rate was 7.38% (95% CI, 1.42-16.75), with high heterogeneity (I^2^ = 90.0%; τ^2^ = 0.0220). Rates ranged from 0% (Miyake^28^) to 33.3% (Rodrigues et al^24^), with the wide range reflecting differences in evaluation methods (photographic vs colorimetric vs clinical assessment), Fitzpatrick skin phototypes of the study populations, and follow-up duration (Fig 3, *D*).

#### Ecchymosis

Three studies reported ecchymosis data. The pooled rate was 22.41% (95% CI, 14.49-31.43), with moderate heterogeneity (I^2^ = 64.9%; τ^2^ = 0.0047). All studies reported ecchymosis as a self-limiting event that resolved without intervention. Rates ranged from 13.0% (Rodrigues et al^24^) to 30.0% (Miyake^28^) (Fig 3, *E*).

### Single-arm meta-analysis: Pooled vessel clearance rate after CLaCS

Five studies provided extractable data on satisfactory vessel clearance and were included in the single-arm meta-analysis. Individual study proportions were 70.59% (95% CI, 56.17-82.51) for Fonseca et al^25^ (36/51); 96.69% (95% CI, 91.75-99.09) for Rosukhovskiy^26^ (117/121); 100.00% (95% CI, 98.13-100.00) for Nasser et al^23^ (195/195); 82.93% (95% CI, 67.94-92.85) for Sathler de Melo and Faccini^29^ (34/41); and 86.43% (95% CI, 79.62-91.63) for Miyake^28^ (121/140). The pooled satisfactory vessel clearance rate after CLaCS was 90.68% (95% CI, 76.74-98.87), with high heterogeneity (I^2^ = 94.6%; τ^2^ = 0.0431; Cochran *Q*, *P* < .0001; Fig 3, *F*).

### Narrative synthesis: CLaCS vs isolated sclerotherapy on vessel clearance

Three studies provided direct comparative data on vessel clearance between CLaCS and isolated sclerotherapy and were synthesized narratively owing to heterogeneity in outcome definitions and study designs. Rosukhovskiy^26^ reported a five-point efficacy score of 3.9 ± 0.6 with CLaCS (LASTIK) vs 2.3 ± 0.8 with compression microsclerotherapy at 1 to 2 weeks (*P* < .0001) and 4.5 ± 0.4 vs 3.8 ± 0.7 at 6 to 10 weeks (*P* < .0001). Nasser et al^23^ reported complete lesion elimination after the third treatment session in 100% of CLaCS-treated patients vs 85.3% of those receiving polidocanol foam sclerotherapy (*P* < .001). Rodrigues et al,^24^ which assessed vessel diameter reduction on B-mode ultrasound rather than photographic clearance, found no significant difference between CLaCS and foam sclerotherapy (62.34% vs 68.45%; *P* = .35). Although the heterogeneity of outcome measures precluded a formal pooled comparative estimate, the qualitative convergence across the two studies that evaluated lesion clearance suggests that CLaCS tends to achieve superior or equivalent vessel elimination compared with isolated sclerotherapy.

### Narrative synthesis: Patient satisfaction

Three studies reported patient satisfaction with treatment outcomes using heterogeneous instruments, precluding statistical pooling. Fonseca et al^25^ used a self-administered 6-level ordinal scale at 30 days postprocedure, with 86.3% of patients rating the result as “excellent” or “very good” (27.5% excellent, 58.8% very good), 11.8% “good,” 2.0% “fair,” and none “poor” or “ineffective.” Rodrigues et al^24^ applied the validated aesthetic numeric analog (ANA) scale (0-10), with mean scores increasing from 4.48 preprocedure to 9.48 postprocedure for CLaCS (*P* < .001) and from 4.62 to 9.14 for foam sclerotherapy (*P* < .001), with no significant difference between techniques. Miyake^28^ reported binary patient-reported outcomes based on before-and-after photographic comparison, with 86% of patients (121/140) classified as having achieved partial or total improvement with patient satisfaction. Despite the methodological heterogeneity, all three studies converged toward consistently high patient satisfaction with CLaCS, in the range of approximately 85% to 90% of patients reporting a satisfactory aesthetic outcome (Table II).

### Risk of bias

**Fig 4:**
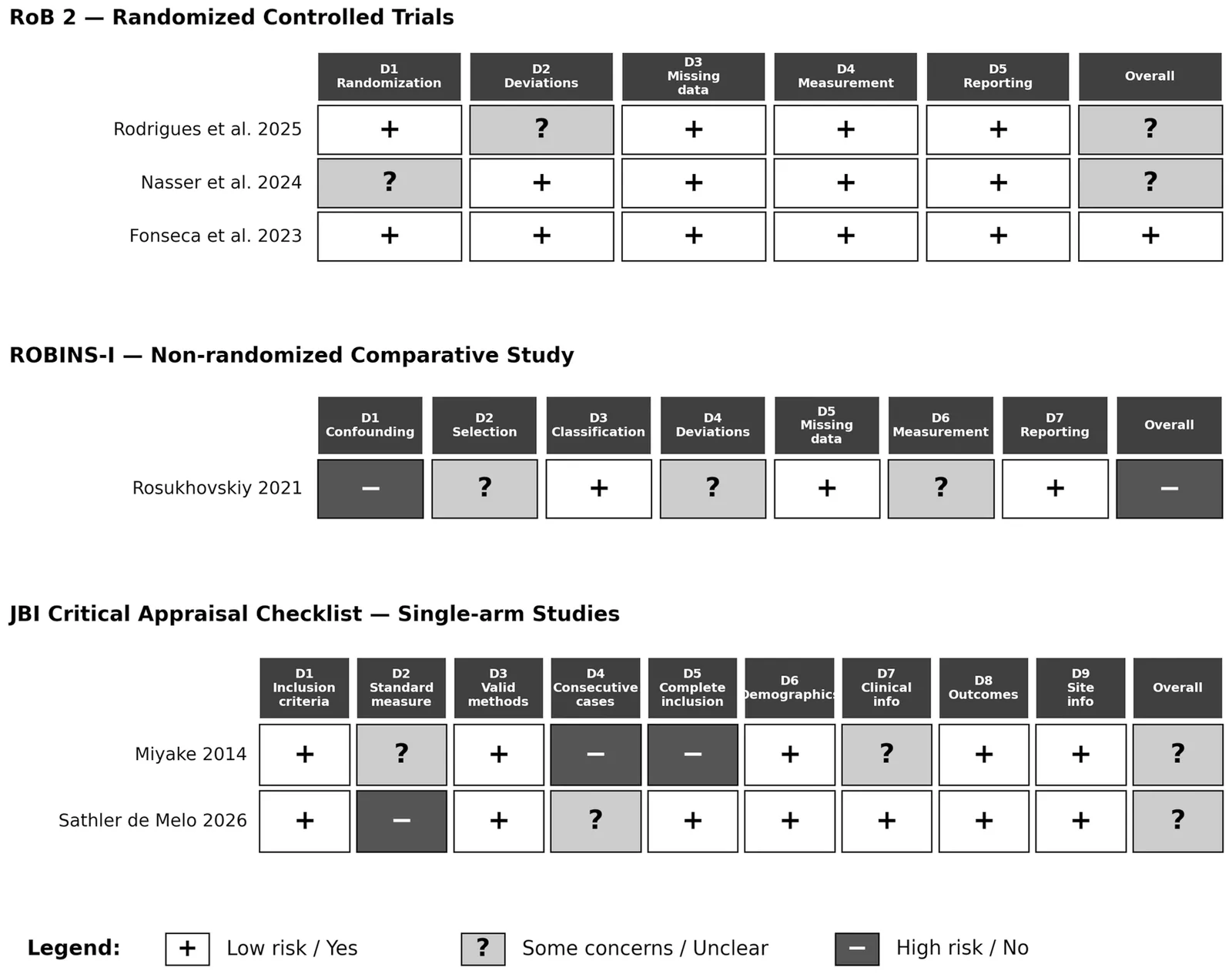
Risk of bias (*RoB*) summary. RoB 2 was applied to randomized controlled trials (RCTS; Rodrigues et al,^24^ Nasser et al,^23^ and Fonseca et al^25^); Risk of Bias in Nonrandomized Studies of Interventions (*ROBINS-I*) was applied to the nonrandomized comparative study (Rosukhovskiy^26^); and the JBI Critical Appraisal Checklist for Case Series was applied to single-arm studies without a comparator (Miyake,^28^ Sathler de Melo, and Faccini^29^). For RoB 2 and ROBINS-I: +, low risk; ?, some concerns; and −, high/serious risk. For JBI: +, Yes; ?, Unclear; and −, No. *JBI*, Joanna Briggs Institute.

Among the three RCTs, Fonseca et al^25^ was judged as low RoB across all domains. Rodrigues et al^24^ and Nasser et al^23^ were judged as having some concerns overall, attributable to the open-label design inherent to comparing visually distinct procedures and, in the case of Nasser et al,^23^ to allocation concealment via sealed envelopes and differential postprocedure protocols between groups. Rosukhovskiy^26^ was assessed using ROBINS-I and judged as serious overall RoB, primarily because of nonrandom allocation by clinic site and unblinded outcome assessment with differential attrition. Miyake^28^ and Sathler de Melo and Faccini^29^ were appraised using the Joanna Briggs Institute Critical Appraisal Checklist for Case Series and rated as having moderate methodological concerns overall. Concerns were identified in both studies regarding standardization and reliability of outcome measurement (Fig 4).

## Discussion

In this systematic review and meta-analysis of six studies encompassing 833 patients, CLaCS was associated with significantly lower rates of telangiectatic matting, microthrombi formation, and ecchymosis compared with isolated sclerotherapy. The pooled incidence of both matting and skin necrosis after CLaCS was near zero. This meta-analysis provides level-II evidence supporting its role as a treatment option with a favorable adverse event profile for lower limb telangiectasias and reticular veins.

The most clinically compelling finding was the near-complete absence of telangiectatic matting after CLaCS (pooled rate, 0.00%; 95% CI, 0.00-0.51), with a 93% relative risk reduction compared with sclerotherapy (RR, 0.07; 95% CI, 0.01-0.36; I^2^ = 39%). Matting represents one of the most concerning adverse events in aesthetic phlebology, with reported incidence rates of 1% to 33% after conventional sclerotherapy.^14^^,^^15^ Its clinical significance extends beyond cosmetic dissatisfaction: matting produces new visible telangiectasias that may worsen the appearance relative to baseline, paradoxically undermining the very goal of treatment and potentially deterring patients from completing their therapeutic plan.^11^ The pathophysiology of matting involves neovascularization triggered by perivascular inflammation, angiogenic mediator release, and arteriolar flow disturbance following sclerosant-induced vessel occlusion.^42^ The near elimination of matting with CLaCS is likely attributable to two synergistic mechanisms. First, the laser-induced endothelial damage and tunica media edema reduce intraluminal diameter before sclerosant injection,^19^ allowing the use of low-concentration, low-volume sclerosants typically hypertonic dextrose, rather than detergent agents, which produce a less aggressive inflammatory cascade.^21^ Second, the high viscosity of dextrose solutions restricts diffusion beyond the target vessel, limiting disruption of surrounding arteriolar networks that would otherwise trigger compensatory neovascularization.^21^

The significant reduction in microthrombi formation (RR, 0.18; 95% CI, 0.06-0.54) is mechanistically coherent with the matting findings and carries direct clinical implications for post-PSH. Microthrombi are a well-established precursor of postinflammatory hyperpigmentation (PIH): trapped intraluminal blood undergoes hemolysis, releasing hemosiderin that deposits in the perivascular dermis, producing the characteristic brown discoloration that may persist for months.^12^^,^^13^ This pathophysiological link was explicitly demonstrated by Rodrigues et al^24^ in their within-patient RCT, where the foam sclerotherapy arm had both significantly more microthrombi (39.1% vs 13.0%; *P* = .035) and higher intensity of pigmentation measured by colorimetry (ΔE 1.44 vs 1.30; *P* = .027). The CLaCS technique appears to attenuate this pathway through two mechanisms: laser-induced vasospasm reduces blood volume within the treated vessel at the time of sclerosant injection,^19^^,^^20^ and the high viscosity of dextrose limits unwanted diffusion into untargeted vessels and capillaries, as demonstrated experimentally by Miyake et al^21^ in an injection pressure model. The clinical implication is that reducing microthrombi may represent the most actionable target for preventing PIH, a complication that, while self-limiting, is the primary driver of patient dissatisfaction after leg vein treatment.^43^

The reduction in ecchymosis (RR, 0.43; 95% CI, 0.32-0.58; I^2^ = 0%) was a remarkably consistent finding across the two reporting studies. This 57% relative risk reduction likely reflects the same hemodynamic advantage: laser-mediated vessel constriction combined with cryoanalgesia-induced superficial vasoconstriction reduces periprocedural blood extravasation.^16^ The pooled single-arm ecchymosis rate of 22.41% (95% CI, 14.49-31.43) indicates that ecchymosis remains the most frequent adverse event associated with CLaCS, although it was uniformly described as self-limiting and clinically insignificant across all studies. This rate compares favorably with the 39% to 46% observed in the sclerotherapy arms of comparative studies^23^^,^^24^ and with the 10% to 30% range reported after conventional sclerotherapy in the broader literature.^44^

The hyperpigmentation analysis warrants careful interpretation. The comparative meta-analysis showed no significant difference under the random-effects model (RR, 0.29; 95% CI, 0.06-1.44), driven by extreme heterogeneity (I^2^ = 92.1%). The directional inconsistency across studies is instructive rather than discouraging: it reveals that the measured outcome depends critically on how hyperpigmentation is defined and assessed. Nasser et al^23^ and Rosukhovskiy^26^ reported substantially lower hyperpigmentation with CLaCS (RR 0.09 and 0.22, respectively), whereas Rodrigues et al^24^ reported a nonsignificant trend in the opposite direction on photographic evaluation (RR, 1.40; 95% CI, 0.53-3.71). Several methodological and population-level factors explain this discordance.

First, the comparator formulations differed: Rodrigues et al^24^ compared CLaCS with liquid dextrose against foam polidocanol, whereas Nasser et al^23^ used liquid dextrose against foam polidocanol and Rosukhovskiy^26^ compared CLaCS against the same sclerosant without laser. Foam formulations inherently produce more microthrombi than liquid agents,^45^ creating distinct sclerosant-tissue interaction dynamics. Second, the study population in Rodrigues et al^24^ had higher Fitzpatrick skin phototypes (60.9% type IV, 8.7% type V), a known independent risk factor for PIH after any dermal procedure.^13^^,^^30^ Notably, in the same study, the subgroup analysis restricted to Fitzpatrick III-IV patients demonstrated a significant advantage for CLaCS in both the number of pigmented limbs (ΔE > 2: 10% vs 35%; *P* = .031) and pigmentation intensity (ΔE, 0.91 vs 1.48; *P* = .006).^24^ Third, and perhaps most importantly, the evaluation method matters: photographic assessment as a binary outcome (present/absent) showed higher rates in the CLaCS arm, whereas the objective colorimetric measurement using the CIELAB color space showed significantly lower intensity of pigmentation with CLaCS.^24^ This dissociation highlights a fundamental limitation of photographic assessment and underscores the potential advantage of quantitative colorimetric methods as proposed by Ly et al^46^ and first applied to postsclerotherapy outcomes by Rodrigues et al^24^ for standardizing PIH evaluation in future comparative studies.

The pooled skin necrosis rate of 0.00% (95% CI, 0.00-0.36) across 595 CLaCS-treated patients is particularly notable given that cutaneous necrosis, although rare, represents the most feared complication of sclerotherapy.^47^ Necrosis typically results from inadvertent injection into or reflux of sclerosant into terminal arterioles, causing ischemic tissue damage.^12^ The near absence of necrosis with CLaCS is plausibly related to the predominant use of hypertonic dextrose as the sclerosing agent. Unlike detergent sclerosants such as polidocanol and sodium tetradecyl sulfate, the high viscosity of dextrose solutions prevents their passage into capillary and arteriolar networks under typical injection pressures, as demonstrated experimentally.^21^ In addition, the reduced volume of sclerosant required in CLaCS Rodrigues et al^24^ reported a median volume of 0.3 mL in the CLaCS arm compared with 2.0 mL in the foam arm (*P* < .0001), further limiting the risk of inadvertent extratarget diffusion. This safety advantage may be particularly relevant in patients with high aesthetic expectations, where even a small necrosis-related scar would be considered a treatment failure.

Beyond safety, our exploratory single-arm meta-analysis demonstrated a high pooled rate of satisfactory vessel clearance after CLaCS (90.68%; 95% CI, 76.74-98.87) across five studies and 548 patients. The estimate is consistent with the individual study results, which ranged from 70.59% in Fonseca et al^25^ to 100% in Nasser et al,^23^ and reinforces the clinical perception that CLaCS achieves vessel elimination at rates comparable to or higher than those reported for conventional sclerotherapy in the broader literature. The high statistical heterogeneity (I^2^ = 94.6%) is expected and largely attributable to differences in how clearance was operationalized across the primary studies namely, blinded photographic assessment with or without augmented reality,^25^ a 5-point efficacy scale,^26^ binary lesion elimination after a fixed number of sessions,^23^ a categorical ≥70% reduction threshold,^29^ and integrated clearance-satisfaction binary outcome^28^ as well as variation in follow-up time points (30 days-12 months). Rather than undermining the pooled estimate, this heterogeneity reflects the absence of a standardized clearance metric in aesthetic phlebology and underscores the need for harmonized outcome definitions in future CLaCS trials.

The narrative comparison of CLaCS vs isolated sclerotherapy on vessel clearance further supports an efficacy advantage that complements its safety profile. Two of three comparative studies that quantified clearance reported superiority of CLaCS: Rosukhovskiy^26^ documented markedly higher 5-point efficacy scores at both early and intermediate follow-up, and Nasser et al^23^ reported complete lesion elimination in 100% of CLaCS-treated patients after three sessions vs 85.3% with foam sclerotherapy. Rodrigues et al,^24^ which used vessel diameter reduction on B-mode ultrasound rather than photographic clearance, found no significant difference between techniques. Although the heterogeneity of designs and outcome metrics precluded a formal pooled comparative estimate, the convergence of two of three studies toward higher clearance with CLaCS is consistent with the proposed synergy between transdermal laser and adjunctive low-aggressiveness sclerotherapy.^19^^,^^48^ This signal should be regarded as hypothesis generating and warrants confirmation in future randomized trials with prespecified, harmonized clearance end points.

Patient satisfaction was reported in three studies using markedly different instruments: a 6-level ordinal categorical scale,^25^ the validated continuous ANA scale,^24^ and a nonvalidated binary patient assessment,^28^ which precluded statistical pooling. Despite this methodological heterogeneity, the three studies converged toward consistently high satisfaction in the range of approximately 85% to 90%, with mean ANA scores reaching 9.48 of 10 after CLaCS in Rodrigues et al.^24^ The agreement of three independent instruments on a similar magnitude of patient-reported benefit reinforces the validity of the finding but also exposes a structural limitation of the field: there is no universally adopted, validated patient-reported outcome measure for aesthetic phlebology. Future trials would benefit from standardizing assessment using a continuous validated scale such as the ANA scale^24^ or the Aberdeen Varicose Vein Severity Score, ideally complemented by objective colorimetric measurement of pigmentation as proposed by Rodrigues et al.^24^ Standardization would enable meaningful pooled estimates and direct cross-study comparison in subsequent meta-analyses.

### Clinical implications

The findings of this review have several implications for clinical practice. For patients at higher risk of PIH, particularly those with Fitzpatrick skin phototypes III through V, who constitute the majority of the population in tropical and subtropical regions. CLaCS may offer a safer alternative to conventional sclerotherapy, with significantly less microthrombi formation and matting. The near-zero matting rate is especially relevant for patients with complex telangiectasias with feeder veins, where conventional sclerotherapy carries the highest risk of this complication. Furthermore, the reduced sclerosant volume required in CLaCS may allow treatment of larger areas in a single session, particularly when the sclerosant is limited to a safe per-session volume,^49^ potentially reducing the number of visits needed for treatment completion.^24^

Our findings align with and provide quantitative support for the 2022 ESVS Clinical Practice Guidelines on the management of CVD, which include CLaCS as a treatment option for C1 disease.^6^ They also complement the 2023 Society for Vascular Surgery, American Venous Forum, and American Vein and Lymphatic Society Clinical Practice Guidelines for the management of varicose veins,^7^ which recommend sclerotherapy as first-line treatment but do not yet formally incorporate CLaCS. The level-II evidence synthesized here may inform future iterations of these guidelines. However, the initial investment in laser equipment limits CLaCS predominantly to private practice settings, and future cost-effectiveness analyses comparing CLaCS with conventional sclerotherapy, factoring in retreatment rates, complication management, and patient satisfaction, would be valuable to inform broader adoption.

### Recommendations for future research

The current evidence base would be substantially strengthened by an adequately powered, multicenter RCT directly comparing CLaCS with isolated sclerotherapy. We propose a two-arm, parallel-group or within-patient (contralateral-limb) design enrolling adults with CEAP C1 telangiectasias and reticular veins, stratified by Fitzpatrick skin phototype (I-III vs IV-VI) given its influence on PIH. The CLaCS protocol should be standardized a priori (Nd:YAG 1064-nm fluence, spot size, pulse duration, cooling method, and sclerosant type and concentration), as should the comparator sclerotherapy regimen. Outcome assessment should be blinded and, wherever possible, objective—colorimetric quantification of pigmentation (CIELAB ΔE), standardized photographic grading of matting and clearance by independent evaluators, and protocolized counting of microthrombi. A coprimary end point structure capturing both safety (eg, hyperpigmentation intensity) and efficacy (eg, blinded vessel clearance) is advisable, with the sample size powered for the comparative pigmentation outcome and a minimum follow-up of 6 to 12 months to capture the natural resolution of pigmentation. Prospective registration and reporting in accordance with the Consolidated Standards of Reporting Trials statement would further improve comparability.

A parallel priority is the development of a standardized core outcome set for the treatment of telangiectasias and reticular veins, ideally endorsed at the society level (eg, by the ESVS and the Society for Vascular Surgery/American Venous Forum). At present, there is no agreed definition or measurement standard for the principal outcomes, which precludes meaningful pooling across studies, as the heterogeneity observed in this review illustrates. Such a core outcome set should specify uniform operational definitions for hyperpigmentation, matting, microthrombi, ecchymosis, and skin necrosis; preferred objective instruments (such as cutaneous colorimetry for pigmentation and blinded photographic assessment for clearance); a validated patient-reported outcome measure, such as the ANA scale; and fixed assessment time points. Adoption of such standards, consistent with the methodology of established core-outcome-set initiatives, would enable direct cross-study comparison and robust future meta-analyses.^50^

### Limitations

This review has several limitations. First, the number of included studies is small (6 studies, with only 3 providing comparative data), which limits the statistical power to detect differences, particularly for rare outcomes such as skin necrosis, and precludes formal publication bias assessment through funnel plots.^40^ Second, heterogeneity was high for hyperpigmentation (I^2^ = 92.1%) and microthrombi (I^2^ = 62.1%) in the comparative analysis and for hyperpigmentation (I^2^ = 90.0%) and microthrombi (I^2^ = 92.4%) in the single-arm analysis, attributable to differences in evaluation methods, sclerosant types, population characteristics, and outcome definitions. Third, although two of three comparative studies were RCTs,^23^^,^^24^ one was a nonrandomized study^26^ conducted at two separate centers with different techniques, introducing potential selection bias and confounding. Fourth, outcome definitions were not standardized across studies: hyperpigmentation was assessed by photographic evaluation,^23^^,^^24^ clinical assessment,^26^ blinded evaluator scoring,^25^ or not precisely quantified,^29^ and microthrombi ranged from intravascular coagula identified on examination^28^ to trapped blood requiring needle thrombectomy.^23^ Fifth, follow-up periods varied considerably (30 days-12 months), and the natural history of adverse events such as hyperpigmentation evolves over months. Nasser et al^23^ reported that the pigmentation rate in the sclerotherapy group decreased from 87.3% at 8 weeks to 45% at 6 months, suggesting that early assessments may overestimate long-term PIH rates. Sixth, ecchymosis data were available from only two comparative studies and three single-arm studies, limiting the robustness of this outcome. Finally, the included studies employed heterogeneous laser parameters (fluence, 50-200 J/cm^2^; pulse duration, 0.65-50 ms; and spot size 2-9 mm), different sclerosant agents, and varying CLaCS protocol implementations, which may affect the generalizability of pooled estimates and underscore the need for standardized technical protocols in future multicenter trials. In addition, patient satisfaction could not be statistically pooled because the three reporting studies used markedly heterogeneous assessment instruments (a 6-level ordinal scale, a continuous validated 0-10 scale, and a nonvalidated binary judgment) and was therefore synthesized narratively. Comparative analysis of CLaCS vs isolated sclerotherapy on vessel clearance was likewise restricted to qualitative synthesis, owing to the limited number of studies (n = 3) reporting clearance comparatively and to differences in their designs, follow-up time points, and outcome metrics, which precluded a formal pooled comparative estimate. Seventh, several outcomes were characterized by very low event counts, with multiple studies reporting zero events; although these studies were retained to preserve information, the Mantel-Haenszel method with a 0.5 continuity correction can overestimate or destabilize treatment effects under such sparse-data conditions, and the corresponding pooled RRs, particularly for matting, microthrombi, and skin necrosis, should therefore be interpreted with caution and regarded as approximate.^35^^,^^36^ Alternative approaches better suited to rare events could not be reliably applied here, given the small number of studies per outcome.^36^ Finally, the evidence base remains small (6 studies, only 3 with comparative data, of which 3 were RCTs), and between-study heterogeneity formally assessed with the Cochran *Q* test and quantified by the I^2^ statistic was substantial for several outcomes; both features limit the precision and certainty of the pooled estimates and temper the strength of the conclusions that can be drawn.

## Conclusions

In this systematic review and meta-analysis of six studies encompassing 833 patients, CLaCS was associated with significantly lower rates of telangiectatic matting (RR, 0.07; 95% CI, 0.01-0.36), microthrombi formation (RR, 0.18; 95% CI, 0.06-0.54), and ecchymosis (RR, 0.43; 95% CI, 0.32-0.58) compared with isolated sclerotherapy for the treatment of lower limb telangiectasias and reticular veins. The pooled incidence of matting and skin necrosis after CLaCS was near zero across all included studies. These findings suggest that CLaCS offers a favorable safety profile, particularly for patients at higher risk of postprocedural pigmentation and matting, such as those with Fitzpatrick skin phototypes III through V, and for those with elevated aesthetic expectations. However, the high heterogeneity observed in hyperpigmentation outcomes and the limited number of comparative studies underscore the need for additional well-designed, multicenter randomized trials with standardized outcome measures, including objective colorimetric assessments and extended follow-up to confirm these findings.

## Author Contributions

Conception and design: GS, DM, SN

Analysis and interpretation: GS, DM, NP, SN, NM

Data collection: GS, AV, JA

Writing the article: GS, DM, AV, JA

Critical revision of the article: GS, NP, SN, NM

Final approval of the article: GS, DM, NP, SN, AV, JA, NM

Statistical analysis: GS

Obtained funding: Not applicable

Overall responsibility: GS

## Declaration of generative AI and AI-assisted technologies in the writing process

During the preparation of this work, the authors used Claude (Anthropic) in order to assist with language editing and improve the readability of the article text. After using this tool, the authors reviewed and edited the content as needed and take full responsibility for the content of the publication.

## Disclosures

None.
