# Supplementary material for: Cryolaser cryosclerotherapy versus isolated sclerotherapy for telangiectasias and reticular veins: A systematic review and meta-analysis
Source: J Vasc Surg Venous Lymphat Disord. 2026 Jul 24;14(6):102582. doi: 10.1016/j.jvsv.2026.102582 (PMC13522362; doi:10.1016/j.jvsv.2026.102582)
Supplement: Supplementary file 1 [file mmc1.docx]

**Supplementary Appendix S1. Full electronic search strategies**

Systematic review and meta-analysis: Cryo-laser cryo-sclerotherapy versus isolated sclerotherapy for telangiectasias and reticular veins.

Databases were searched from inception through February 2026. No language or publication-date restrictions were applied. Reference lists of the included studies and of relevant reviews were hand-searched for additional eligible records. Records retrieved: PubMed/MEDLINE, n = 28; Embase, n = 48; Cochrane CENTRAL, n = 6 (total, 82; 36 duplicates removed, leaving 46 unique records screened).

Because cryo-laser cryo-sclerotherapy is a specific, named intervention, the search was built around the intervention concept and combined, where appropriate, with a laser concept. Search-set syntax is presented in the native format of each platform.

**1. PubMed / MEDLINE**

#1 “cryo laser cryo sclerotherapy” OR “cryo-laser cryosclerotherapy” OR cryosclerotherapy OR cryolaser OR “cryo laser” OR CLaCS

#2 “Lasers, Solid-State”[Mesh] OR laser OR “Laser Therapy”[Mesh] OR “laser therapy” OR “Nd:YAG” OR “1064 nm” OR transdermal

#3 #1 AND #2

**2. Embase**

#1 ‘cryo laser cryo sclerotherapy’ OR ‘cryo-laser cryosclerotherapy’ OR cryosclerotherapy OR cryolaser OR ‘cryo laser’ OR clacs

#2 ‘laser’/exp OR laser OR ‘laser therapy’/exp OR ‘laser therapy’ OR ‘nd:yag’ OR ‘1064 nm’ OR transdermal

**3. Cochrane Central Register of Controlled Trials (CENTRAL)**

#1 (“cryo-laser cryo-sclerotherapy” OR “cryo-laser cryosclerotherapy” OR cryosclerotherapy OR cryolaser)
